# Emerging trends and research hot spots in inborn error of immunity: A bibliometric perspective

**DOI:** 10.1016/j.jacig.2026.100704

**Published:** 2026-04-15

**Authors:** Hiba Alblooshi, Muhamad Jalal Khan, Farida Almarzooqi

**Affiliations:** a Department of Genetics and Genomics, College of Medicine and Health Sciences, United Arab Emirates University, Al Ain, United Arab Emirates; b Department of Internal Medicine, College of Medicine and Health Sciences, United Arab Emirates University, Al Ain, United Arab Emirates

**Keywords:** Inborn errors of immunity, bibliometrics, genomics, precision medicine, global collaboration, immunodeficiency, rare diseases, translational immunology, multiomics, health equity

## Abstract

**Background:**

Inborn errors of immunity (IEIs) are an expanding group of genetically defined disorders associated with infections, autoimmunity, and malignancy. Advances in high- throughput genomics and updates to international classifications have reformed the field, shifting from phenotype-based descriptions to molecular frameworks. Bibliometric analysis offers a structured approach to mapping research growth, collaboration, and thematic evolution.

**Objective:**

We analyzed global IEI research from 1995 to 2025 using bibliometric methods, focusing on publication trends, collaboration networks, leading contributors, and thematic shifts.

**Methods:**

Publications were retrieved from Web of Science Core Collection and PubMed. After screening, 840 original articles were analyzed with Biblioshiny and VOSviewer to assess citation patterns, coauthorship, thematic clusters, and keyword evolution.

**Results:**

IEI research (n = 840; 333 journals; 7,466 authors) increased at 4.5% annually, with a marked rise after 2018 after next-generation sequencing and International Union of Immunologic Societies classification updates. The United States produced the largest output, while European countries had a higher citation impact per article. Collaboration was strongest between North America and Europe, with other regions remaining more domestically focused. Thematic mapping revealed a transition from clinical phenotypes to genetic, multiomic, and precision frameworks, alongside growing focuses on autoinflammation, immune dysregulation, and rare disease subgroups.

**Conclusion:**

Over 3 decades, IEI research has expanded substantially, reflecting a paradigm shift toward molecular discovery and international collaboration. Persistent regional disparities highlight the need for inclusive genomic studies and equitable partnerships, providing strategic insights to advance clinical immunology and enhance care for patients with rare immune disorders.

Inborn errors of immunity (IEIs), formerly referred to as primary immunodeficiency, represent an expanding group of genetically defined disorders marked by immune dysregulation, susceptibility to infection, autoimmunity, and malignancy. Recent advances in high-throughput genomics and updated international classification systems have accelerated the discovery of novel pathogenic variants and mechanisms, broadening our understanding of the clinical complexity of IEIs.^1^, ^2^, ^3^, ^4^, ^5^

In this rapidly evolving landscape, bibliometric analysis offers a systematic approach to examining the intellectual structure and development of scientific fields. Bibliometrics helps us understand research activity by analyzing publication counts, citation patterns, coauthorship links, and keyword trends. This reveals how productive researchers are, how they collaborate globally, what new topics are emerging, and where gaps exist in the current knowledge.^6^^,^^7^ These understandings are particularly relevant to IEIs, as international efforts such as International Union of Immunologic Societies (IUIS) continue to revise and expand disease classifications in response to rapid scientific and genomic advances.^1^

Previous studies on IEIs have largely focused on historical overviews, genetic discoveries, and evolving clinical phenotypes, particularly in relation to advances in immunogenetics and next-generation sequencing.^2^ These reviews provide important qualitative perspectives on the development of the field, but they do not quantitatively assess research output or publication trends. To our knowledge, no dedicated bibliometric analysis of IEIs has been published; therefore, we conducted this study to provide the first systematic bibliometric evaluation of IEI research from 1995 to 2025. We analyzed publication trends, collaboration networks, key contributors, thematic evolution, and knowledge gaps; our results will help inform future research directions and translational strategies.

## Methods

### Publication search

**Fig 1:**
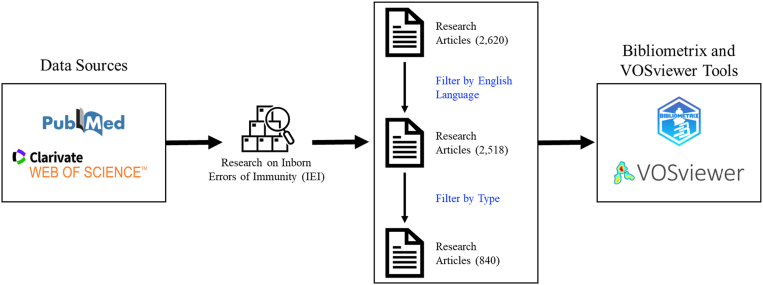
Flowchart showing retrieval strategy. Publications on IEIs were retrieved from PubMed and WoSCC. From 2,620 records identified, 2,518 English-language articles were screened, yielding 840 original studies for analysis with Biblioshiny and VOSviewer to evaluate citation patterns, collaboration networks, and thematic trends.

We carried out comprehensive searches in the Web of Science Core Collection (WoSCC; www.webofscience.com) and PubMed (pubmed.ncbi.nlm.nih.gov) databases to identify research on IEIs. The search covered the period from January 15, 1995, to January 15, 2025, and the data were retrieved on January 20, 2025. The search parameter flowchart is shown in Fig 1, with further details provided in Table E1 in the Online Repository available at www.jaci-global.org. From WoSCC, 1,236 articles were identified, with 750 studies met the language and relevance criteria. From PubMed, 1,384 articles were identified, with 630 studies meeting the language and relevance criteria. A total of 540 articles were common to both databases, while 210 articles were unique to WoSCC and 90 articles were unique to PubMed. Therefore, a total of 840 articles were included in this study.

### Rationale for choosing WoSCC

The WoSCC was selected for this study because of its broad coverage of peer-reviewed journals and its proven track record of delivering reliable bibliometric data for academic research. It provides robust citation analysis tools, which are invaluable for tracking research trends and patterns. The WoSCC tools also offer accurate metadata and systematic indexing, which contribute to bibliometric evaluations. However, to ensure a more comprehensive and balanced representation of the literature, WoSCC was supplemented with data from PubMed, which provides a wider range of biomedical literature. This includes articles from journals not indexed in WoSCC. By utilizing complementary databases, we were able to perform a more inclusive and robust analysis. Therefore, both WoSCC and PubMed provide a reliable foundation for bibliometric research in medical domains.

### Inclusion and exclusion criteria

We included research articles published in the English language that focused on IEIs from both WoSCC and PubMed. All selected articles underwent manual screening and expert evaluation to ensure their relevance and quality. For exclusion, we omitted publications such as review articles, book chapters, conference proceedings, meeting abstracts, editorial materials, letters, news items, and retracted papers. In addition, we excluded articles published in languages other than English, those not indexed in WoSCC (for the PubMed database), and any that expert assessment deemed irrelevant.

### Literature screening process

We conducted a systematic screening procedure to identify relevant literature on IEIs. An initial search across both WoSCC and PubMed yielded a total of 2,620 publications. To retain only English-language publications, a language filter was applied that resulted in 2,518 articles for title and abstract screening. During this phase, articles that did not align with the predefined inclusion criteria were excluded. A total of 1,678 records were removed for the following reasons: 682 were excluded on the basis of domain expert evaluation, 540 were duplicate records from PubMed, 354 were review articles, 24 were missing in WoSCC, 22 were book chapters, 21 were conference proceedings, 19 were meeting abstracts, 11 were editorial materials, 3 were letters, 1 was a news item, and 1 was a retracted paper. Therefore, our comprehensive screening process ensured the relevance and quality of the final selection. In the end, we included 840 articles in the final dataset: 540 articles found in both databases, 210 unique research articles from WoSCC, and 90 unique articles from PubMed (indexed in WoSCC), as shown in Table E1.

### Data analysis

We utilized data analysis tools such as the Biblioshiny platform (www.bibliometrix.org/home/index.php/layout/biblioshiny) and VOSviewer (www.vosviewer.com) to conduct and visualize the bibliometric analysis.^8^^,^^9^ Biblioshiny is a web interface for the ‘bibliometrix’ R package, and we used it to produce visual outputs such as trending topics, thematic maps, and global distribution charts.^8^ For the quantitative evaluation and management of publication data, we relied on Microsoft Excel to ensure precision and consistency. Similarly, VOSviewer facilitates the construction and visualization of bibliometric networks to examine contributions from countries, institutions, journals, authors, keywords, and individual publications.^9^ We used these networks to illustrate relationships based on citation links, cocitation patterns, bibliographic coupling, and coauthorship.

## Results

### Publication trends and output growth

**Fig 2:**
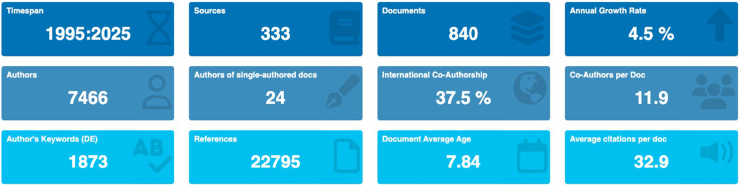
Characteristics of IEI literature from 1995 to 2025. Main bibliometric indicators included IEI literature, generated by Biblioshiny platform. Metrics include publication count, authorship patterns, collaboration rates, citation impact, and document characteristics.

The IEI-related publications from 1995 to 2025 compromised 840 documents sourced from 333 journals, authored by 7,466 researchers (Fig 2). This reflects substantial scholarly engagement in the field. The average annual growth rate of 4.5% indicates a consistent rise in research activity over time. The dataset reveals strong collaborative patterns, with an average of 11.9 coauthors per document and 37.5% international coauthorship, highlighting the global nature of IEI research. A total of 22,795 references and 1,873 author-defined keywords were recorded, contributing to the field’s thematic breadth. The average document age is 7.8 years, and the mean citation count stands at 32.9, suggesting a moderate to high level of academic influence. All data were generated by the Biblioshiny platform.

**Fig 3:**
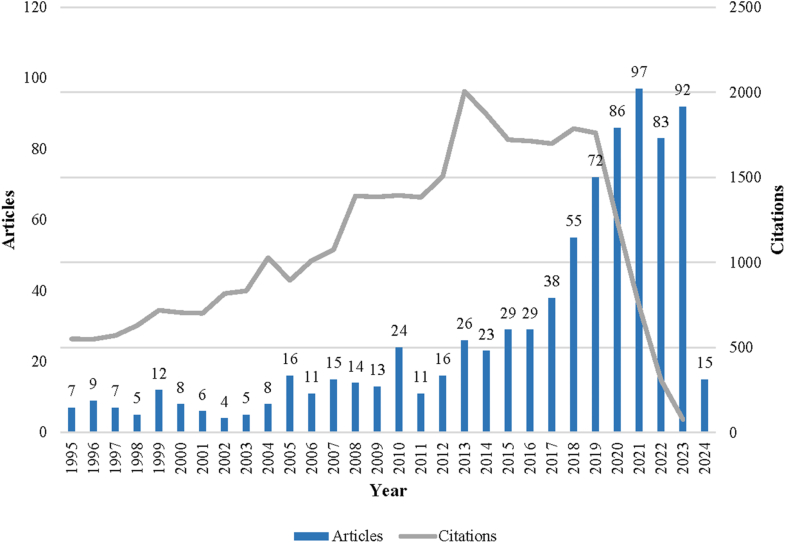
Annual trends in number of IEI publications and citations from 1995 to 2025. Publications are represented by *columns;* citations are shown as *lines* plotted on secondary axis to reflect differing magnitudes.

The annual publication and citation trends showed a steady increase in IEI-related publications from 1995 onward, with a notable surge beginning in 2018 and peaking in 2021 (Fig 3). Citations rose accordingly, reaching their highest point in 2019, before declining slightly, likely because of the recentness of newer publications. This trend reflects the growing global interest and impact of IEI research over the past decades.

### Global collaboration landscape

**Fig 4:**
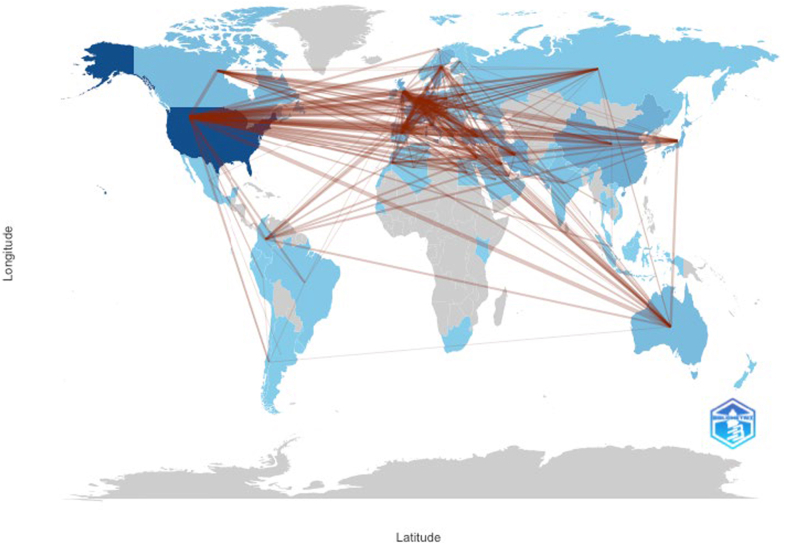
Global collaboration network in IEI research. Visualizes international collaboration patterns in IEI research. Each *line* represents coauthorship links between countries, with *line density* reflecting strength and frequency of collaboration; *darker nodes* indicate higher research output.

The global landscape of collaborative research in IEIs highlighting strong transnational ties between North America, Europe, and Asia. The United States, as a central node, demonstrates extensive collaborative links with European countries such as France, Germany, and the United Kingdom, as well as with nations in the Middle East, East Asia, and Oceania (Fig 4). This extensive collaborative network highlights the inherently global and multidisciplinary character of IEI research, wherein complex clinical and molecular investigations are advanced through international cooperation and the integration of diverse scientific expertise. Among the top 10 countries contributing to research on IEIs is the United States, which led with the highest number of publications (n = 1,307) and citations (n = 10,105), followed by Iran (n = 507) and France (n = 470) (see Table E2 in the Online Repository available at www.jaci-global.org). England showed the highest average citations per publication (77.6), reflecting strong research impact (quality of publications), followed by Germany (68.2) and France (54.9). The analysis also revealed top contributing institutions, including Université Paris Cité, Institut National de la Santé et de la Recherche Médicale (INSERM), and Assistance Publique–Hôpitaux de Paris (AP-HP) from France; the National Institutes of Health and Harvard University from the United States; and the Tehran University of Medical Sciences (Iran), the University of Freiburg (Germany), and the University of London (United Kingdom). These findings highlight key national and institutional contributions to IEI research, with North America and Western Europe institutions demonstrating higher citation impact.

**Fig 5:**
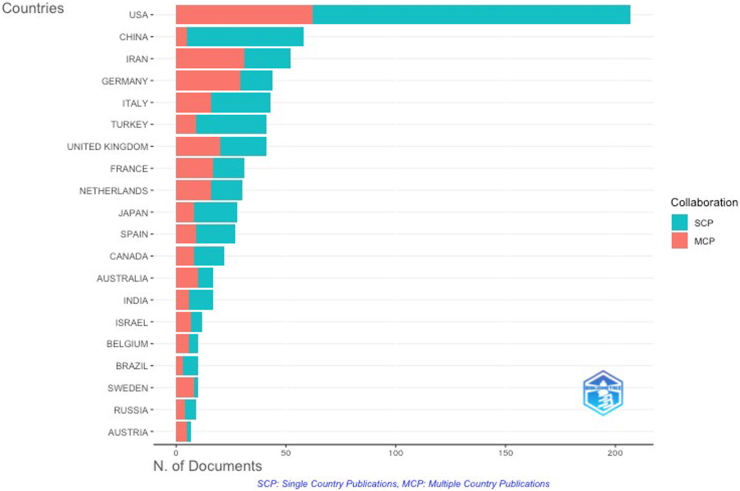
Geospatial map illustrating countries of corresponding authors. IEI-related publications shown according to corresponding authors’ countries, categorized into single-country publications (SCP) and multicountry publications (MCPs). SCPs represent national efforts, while MCPs reflect international collaboration.

The United States leads IEI research output by corresponding authors, with a substantial proportion involving international collaboration (Fig 5). Other major contributors include China, Iran, Germany, and Italy, each showing a mix of national and international publications. Notably, countries such as China and Iran have a relatively higher proportion of domestic (single-country) publications, whereas European countries and the United States show a stronger tendency toward international collaboration (multicountry publications). These patterns highlight both the global reach and varying degrees of cross-border cooperation in IEI research leadership. Further international coauthorship patterns among countries and organizations, revealing densely connected collaborative networks that align with leading research outputs, are illustrated in Figs E1 and E2 in the Online Repository available at www.jaci-global.org.

### Leading authors’ influence

The most productive and influential authors in the field of IEIs are Nima Rezaei, Luigi Notarangelo, and Hassan Abolhassani; each contributed 27 publications, with Rezaei having the highest citation count among them (n = 1,523) (see Table E3 in the Online Repository available at www.jaci-global.org). Jean-Laurent Casanova and Capucine Picard demonstrated notable scholarly impact, particularly Picard, who attained the highest citation total (n = 4,261) and H-index (17), reflecting sustained and high-quality contributions to the field. These investigators have been instrumental in elucidating the molecular and genetic basis of IEIs, particularly in defining novel monogenic defects and their immunopathogenic mechanisms. Cocitation analysis further identified Charlotte Cunningham-Rundles, Capucine Picard, and Waleed Al-Herz as highly cocited authors, underscoring their conceptual influence and the integration of their work across diverse IEI studies. Frequent coauthorship patterns such as between Casanova and Picard, or Rezaei and Abolhassani, highlight the collaborative and interdisciplinary structure of IEI research, which spans clinical immunology, molecular diagnostics, and human genetics. Network visualizations further support these findings (see Fig E3 in the Online Repository).

### Core journals and key references

The top journals contributing to IEI literature are *Frontiers in Immunology,* which ranked highest in publication count (n = 75), followed by the *Journal of Clinical Immunology* (n = 69) and the *Journal of Allergy and Clinical Immunology* (n = 48), all Q1-ranked journals (Table I). Despite lower article counts, *Blood* and the *Journal of Clinical Investigation* demonstrated high citation impact (impact factors of 21 and 13.3, respectively). Cocitation analysis identified the *Journal of Allergy and Clinical Immunology* as the most frequently cocited source (n = 2,445), followed by *Blood* (n = 1,998) and the *Journal of Clinical Immunology* (n = 1,673), reflecting their pivotal role in disseminating specialized, field-relevant research and serving as core references for immunologists working on IEIs (Table II). These journals consistently publish both clinical and mechanistic studies, which bridge diagnostic, therapeutic, and immunopathogenic insights. Notably, the frequent cocitation of high-impact journals such as *Nature, Science,* and the *New England Journal of Medicine* highlights the strong interface between clinical immunology and basic molecular science. This suggests that advances in IEIs often emerge from integrative research that connects molecular and genetic mechanisms with clinical phenotypes, thereby reinforcing the translational nature of the field. Citation and cocitation networks, along with overlay visualizations of journals, highlight the thematic structure, influence, and foundational literature in IEI research (see Fig E4 in the Online Repository available at www.jaci-global.org).

**Table I:** Leading journal sources in IEI literature

No.	Journal	Counts	Citations	IF	Q
1	*Frontiers in Immunology*	75	1926	5.7	Q1
2	*Journal of Clinical Immunology*	69	2833	7.2	Q1
3	*Journal of Allergy and Clinical Immunology*	48	1970	11.4	Q1
4	*Blood*	21	1684	21	Q1
5	*Frontiers in Pediatrics*	20	85	2.1	Q2
6	*Clinical Immunology*	19	189	4.5	Q2
7	*Iranian Journal of Allergy Asthma and Immunology*	13	41	1.2	Q4
8	*Journal of Clinical Investigation*	13	780	13.3	Q1
9	*Allergologia et Immunopathologia*	11	50	2.1	Q3
10	*Clinical and Experimental Immunology*	11	120	3.4	Q2

Top 10 journals publishing research on IEIs, ranked by article count, along with total citations, impact factors (IF), and quartile rankings (Q).

**Table II:** Leading cocited sources in IEI literature

No.	Cocited journal	No. of cocitations	IF	Q
1	*Journal of Allergy and Clinical Immunology*	2445	11.4	Q1
2	*Blood*	1998	21	Q1
3	*Journal of Clinical Immunology*	1673	7.2	Q1
4	*Frontiers in Immunology*	921	5.7	Q1
5	*Science*	871	44.7	Q1
6	*Nature*	832	50.5	Q1
7	*Journal of Experimental Medicine*	805	12.6	Q1
8	*New England Journal of Medicine*	772	96.2	Q1
9	*Proceedings of the National Academy of Sciences of the United States of America (PNAS)*	720	11.1	Q1
10	*Journal of Immunology*	656	4.5	Q2

Top 10 most frequently cocited journals (reflecting field-influencing sources) publishing research on IEIs, ranked by article count, along with total citations, impact factors (IF), and quartile rankings (Q).

The most cited reference was the 2019 IUIS classification update by Tangye et al (n = 100), followed by the American College of Medical Genetics and Genomics/Association for Molecular Pathology variant interpretation guidelines by Richards et al (n = 71) and the 2017 IUIS classification report by Picard et al (n = 57). The foundational references most frequently cocited in IEI literature are listed in Table E4 in the Online Repository available at www.jaci-global.org. These documents collectively provide essential frameworks for genetic variant interpretation and immunodeficiency classification. Other highly cited works include European Society for Immunodeficiencies diagnostic definitions, newborn screening outcomes, and landmark studies on *CTLA4* (cytotoxic T-lymphocyte–associated protein 4) variants and transplantation in the context of severe combined immunodeficiency. The frequent cocitation of both consensus guidelines and mechanistic studies underlines the dual reliance on standardized frameworks and molecular understandings within the IEI research community. The cocitation network of these references is presented in Fig E5 in the Online Repository.

### Keyword trends and thematic development

**Fig 6:**
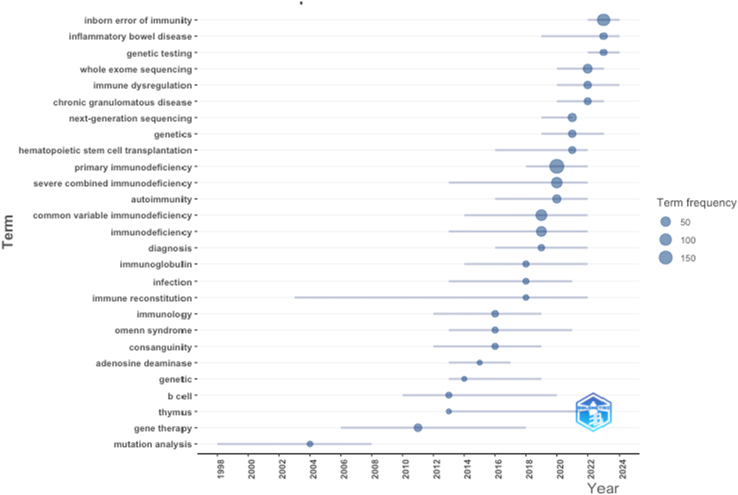
Trend topics by author keywords in IEI research from 1995 to 2025. Each term is mapped along timeline according to its first and last appearance, with *bubble size* representing its frequency of use. *Larger bubbles* indicate higher prevalence, while *horizontal lines* reflect duration over which each term remained active in literature. Terms like “inborn error of immunity,” “genetic testing,” and “immune dysregulation” have emerged as dominant topics in recent years, while foundational terms such as “severe combined immunodeficiency/SCID,” “gene therapy,” and “immunoglobulin” have seen declining prominence.

Analysis of trend topics reveals a thematic transition in IEI research, shifting from classical immunologic concepts to molecular and genomic frameworks (Fig 6). Post-2018 publications increasingly emphasize keywords such as “inborn error of immunity,” “genetic testing,” and “whole-exome sequencing,” reflecting the adoption of precision diagnostics. Meanwhile, persistent use of terms like “primary immunodeficiency,” “autoimmunity,” and “hematopoietic stem cell transplantation” suggests continued relevance of clinical management topics. Notably, the emergence of “inborn errors of immunity” as a dominant keyword reflects a conceptual shift in the field, replacing the narrower term “primary immunodeficiency” to encompass a more comprehensive understanding that includes monogenic immunodysregulation, autoinflammation, and susceptibility to infection and malignancy. This pattern underlines the integration of next-generation sequencing and molecular techniques into the diagnostic and therapeutic makeup of IEIs. Keyword co-occurrence and recency overlay analyses support this shift (see Fig E6 in the Online Repository available at www.jaci-global.org).

**Fig 7:**
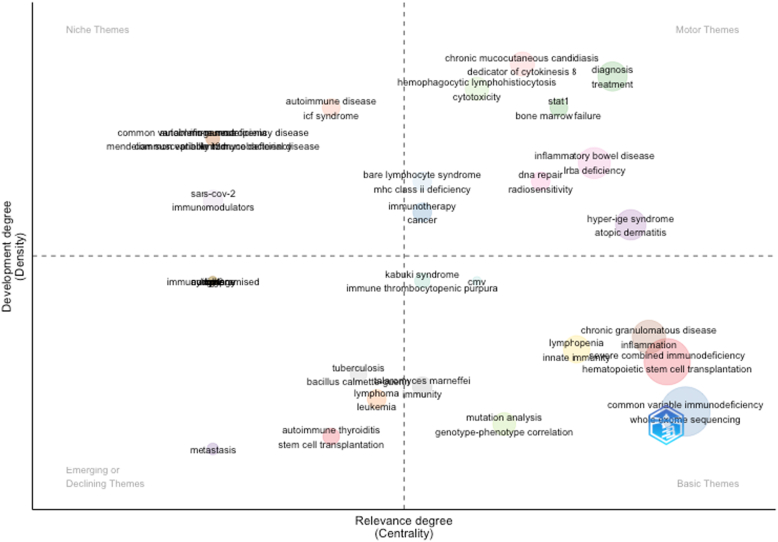
Thematic map of author keywords in IEI research. *Bubbles* represent themes, positioned by centrality (x-axis, relevance to field) and density (y-axis, internal development). *Bubble size* reflects publication volume. *Quadrants* indicate motor (central, developed), niche (developed, isolated), basic/transversal (central, emerging), and emerging/declining (peripheral, underdeveloped) themes.

The thematic map in Fig 7 highlights key research trends in IEIs that are based on keyword analysis. Among the most central and foundational themes were “common variable immunodeficiency,” “severe combined immunodeficiency,” “whole-exome sequencing,” and “hematopoietic stem cell transplantation,” all located in the lower right quadrant, indicating high relevance but requiring further development. These themes are central to immunodeficiency research and represent core clinical concerns. In the upper right quadrant, motor themes such as “chronic mucocutaneous candidiasis,” “hemophagocytic lymphohistiocytosis,” “diagnosis,” and “treatment” showed high centrality and density, reflecting their established and dynamic role in current research. Keywords like “STAT1” (signal transducer and activator of transcription 1), “inflammatory bowel disease,” and “hyper-IgE syndrome” also clustered here, signifying active investigation and clinical significance. Niche themes (upper left), including autoimmune diseases (eg, autoimmune neutropenia), ICF (immunodeficiency, centromeric instability, and facial anomalies) syndrome, and Mendelian susceptibility to mycobacterial diseases, appeared well developed but less integrated with the broader thematic network. These may reflect specialized or advanced subfields. In contrast, emerging or declining themes such as “metastasis,” “immunocompromised,” and “SARS-CoV-2” (severe acute respiratory syndrome coronavirus 2) appeared in the lower left quadrant. Their positioning may suggest limited development or decreasing relevance within the context of IEIs, although further investigation may clarify their trajectory. This analysis supports the identification of research hot spots, underexplored areas, and potential directions for future studies within the domain of IEIs.

## Discussion

### Genomic advances and evolving concepts in IEIs

The consistent rise in IEI-related publications from 1995 to 2025, with a marked surge after 2018, reflects a transformative period in immunology, driven by the integration of next-generation sequencing and precision diagnostics. The emergence of terms like “whole-exome sequencing” and “inborn errors of immunity” confirms a conceptual shift from classical phenotypic classification to genetically defined disease frameworks.^2^^,^^10^ Previous studies highlight that multiomic platforms help overcome key limitations of single-modality genomics in iridiagnostics. While whole-exome sequencing has advanced the identification of monogenic Irishman, cases remain unresolved as a result of variants of uncertain significance or noncoding regions.^11^, ^12^, ^13^ Integrating transcriptomic, proteomic, and metabolomic data improves variant interpretation and enhances clinical relevance.^2^^,^^11^ Thus, multiomic approaches will improve variant interpretation and support clinical decisions, especially in critically ill and pediatric patients, and may serve as a pivotal foundation for the future of molecularly defined precision immunology.

### Global collaboration and clinical inequities

Our bibliometric analysis highlights both progress and disparity in international IEI research. The United States, France, and the United Kingdom dominate multicountry collaborations, while countries such as China and Iran show strong but largely domestic publication outputs (single-country publications).^14^ Although several European nations publish fewer articles overall, their higher citation-per-publication ratios suggest that visibility and collaboration density often translate into greater clinical impact.^15^ These disparities have real-world consequences. Diagnostic inequities, particularly among ethnic minorities and in low- and middle-income countries, lead to prolonged diagnostic odysseys, with delayed treatment initiation and adverse outcomes.^16^^,^^17^

While registries such as the European Society for Immunodeficiencies, encompassing >30,000 patients, offer unprecedented opportunities for outcome monitoring, their success depends on equitable data sharing and broad geographic participation.^16^^,^^18^ Initiatives such as the J Project have expanded diagnostic access in Eastern and Central Europe, but significant gaps remain in resource-limited settings.^16^^,^^19^ High consanguinity rates in many low- and middle-income countries exacerbate the prevalence of autosomal-recessive IEIs, yet limited genetic infrastructure and translational research centers perpetuates underdiagnosis.^20^ Addressing these disparities requires not only scientific advances but also intentional structural changes. Patient registries, rare disease research networks, and interoperable data frameworks have demonstrated that coordinated investment can expand access to genomic diagnostics and therapies.^21^^,^^22^ Partnerships between academia, regulators, and patient communities, supported by industry, are particularly effective in accelerating translation, aligning ethical trial design, and enhancing real-world evidence generation.^23^^,^^24^ To ensure that discoveries benefit all patient populations, inclusive funding strategies and harmonized governance policies are needed.^25^ Within the Middle East and North Africa (MENA) region, emerging programs in Gulf Cooperation Council countries and North Africa exemplify how targeted investment can address these structural barriers and advance understanding of region-specific genetic architecture. Saudi Arabia’s implementation of comprehensive newborn screening programs, including T-cell–receptor excision circle–based severe combined immunodeficiency detection combined with next-generation sequencing, has revealed incidence rates higher than previously documented, indicating both the feasibility and necessity of early diagnostic infrastructure in consanguineous populations.^26^^,^^27^ Large-scale genomic initiatives in Qatar, particularly through the Qatar Biobank and Qatar Genome Programme, have further characterized the genetic landscape of autoinflammatory diseases and quantified the burden of Mendelian disorders in Middle Eastern populations, revealing variant spectra distinct from European cohorts and underscoring the need for population-specific reference datasets.^28^^,^^29^ Complementary efforts the United Arab Emirates have used next-generation sequencing to characterize major histocompatibility complex diversity, providing critical immunogenetic baseline data for transplantation medicine and autoimmune disease research in the region.^30^ In North Africa, the MENA-IEI Registry Network represents a transformative model of multinational collaboration, encompassing 17,120 patients from 22 countries and with an 83% genetic diagnostic yield through coordinated phenotypic and molecular characterization.^31^ The Institut Pasteur International Network in Tunisia, Algeria, and Morocco highlights how international collaboration can reduce disparities in access to precision medicine through capacity building and shared expertise by enabling local genetic testing for common primary immunodeficiencies and partnering with centers in high-income countries for complex cases.^32^ These programs provide replicable models for improving access to evidence-based immunodeficiency care in resource-variable settings.^33^ Stronger collaboration within the MENA region and with high-resource centers in North America, Europe, and Asia is urgently needed to enable technology transfer, joint clinical trials, training, and equitable access to emerging gene and cell therapies that remain largely unavailable locally.^32^^,^^34^ Obtaining global equity in rare disease care will require multilateral partnerships that prioritize harmonized governance and inclusive funding to ensure innovations benefit patients regardless of geography.^35^^,^^36^

### Mentorship and capacity building in IEI research

The field remains shaped by a small number of high-output centers such as the US National Institutes of Health, Université Paris Cite, and Tehran University, which dominate scholarly impact. While this fosters scientific excellence, it risks limiting opportunities for early-career investigators and institutions in underrepresented regions.^15^ Inclusive mentorship pipelines, exemplified by the J Project and IUIS training initiatives, demonstrate the value of cross-border mentorship models.^14^^,^^37^ These programs provide exposure to rare clinical cases, participation in international discussions, and access to large-scale datasets.^38^ Bridging conventional postgraduate training with global mentorship networks will be critical to building adverse workforce capable of advancing clinical immunology.^39^, ^40^, ^41^

### Strategic value of bibliometric intelligence

Bibliometric mapping offers more than descriptive insights; it can guide clinical and translational strategy. The identification of core journals, guideline anchors (IUIS, American College of Medical Genetics and Genomics/Association for Molecular Pathology), and high-impact authors reflects the consolidation of knowledge dissemination pathways. Notably, our analysis revealed underexplored areas such as health-related quality of life, which remain insufficiently represented despite their recognized importance to patient-centered outcomes.^1^^,^^7^ Additionally, bibliometric approaches can systematically reveal field-level and translational gaps that are not routinely captured by hypothesis-driven research, including geographic disparities, imbalance between gene discovery and functional validation, delayed translation into clinical guidelines, and limited focus on psychosocial and implementation outcomes.^2^^,^^5^^,^^42^, ^43^ Looking ahead, the trajectory of IEI research will be shaped by the integration of human expertise, genomic technologies, and artificial intelligence. Emerging platforms such as GenIA demonstrate potential in variant prioritization and early diagnostic prediction, although challenges related to harmonization and algorithmic equity persist.^38^^,^^43^, ^44^, ^45^, ^46^ The integration of bibliometric intelligence with genomics and artificial intelligence–driven approaches offers an opportunity to close knowledge gaps, accelerate translational advances, and optimize global research strategies.

### Conclusion

This bibliometric analysis offers a comprehensive overview of the scientific trajectory of IEIs over 3 decades, highlighting both the field’s expansion and its transformation in scope and focus. The results demonstrate a sustained rise in research activity, particularly after the advent of next-generation sequencing and major updates to IEI classification systems, reflecting a paradigm shift toward molecularly driven diagnostics and therapies. The mapping of global collaboration patterns reveals a vibrant yet uneven international research ecosystem, with high-output countries like the United States and France fostering extensive cross-border networks, while emerging contributors such as Iran and China remain largely domestically focused. Underrepresented countries contributed fewer studies, showed weaker integration into global networks, and faced limited access to genomic tools, underscoring persistent inequities in visibility and clinical impact. These findings emphasize the importance of enhancing equitable international partnerships, especially to address rare disease challenges that require large, diverse datasets. Furthermore, the identification of high-impact authors, institutions, and foundational literature reflects the concentration of scholarly influence and the importance of conceptual continuity in shaping the field. The cocitation of consensus guidelines alongside mechanistic studies illustrates the integrative nature of IEI research, which bridges clinical practice and basic science. Finally, the use of bibliometric intelligence has proven valuable not only in tracing the field’s development but also in identifying emerging themes, underexplored niches, and strategic opportunities for innovation. The insights gained from this study can support evidence-based research planning, promote inclusive scientific growth, and guide the next generation of discoveries in immunogenetics and clinical immunology.

**Clinical implication**

Expanding genomic research and ensuring equitable access to diagnostics are essential to reduce diagnostic delays and improve outcomes for patients with immunologic disorders.

### Declaration of generative AI and AI-assisted technologies in the writing process

The authors used ChatGPT (OpenAI) for language editing and reorganization. All content was reviewed and edited by the authors, who take full responsibility for the final report.

## Disclosure statement

Disclosure of potential conflict of interest: The authors declare that they have no relevant conflicts of interest.

## Supplementary data

Supplementary Figs E1-E6

Supplementary Tables E1-E4

## References

[1] Tsoulis M.W., Williams K.W. (2025). Keeping up with recent developments in immunodeficiency. Ann Allergy Asthma Immunol.

[2] Akalu Y.T., Bogunovic D. (2024). Inborn errors of immunity: an expanding universe of disease and genetic architecture. Nat Rev Genet.

[3] Bucciol G., Delafontaine S., Meyts I., Poli C. (2024). Inborn errors of immunity: a field without frontiers. Immunol Rev.

[4] Ren A., Yin W., Miller H., Westerberg L.S., Candotti F., Park C.S., Lee P. (2021). Novel discoveries in immune dysregulation in inborn errors of immunity. Front Immunol.

[5] Hurabielle C., LaFlam T.N., Gearing M., Ye C.J. (2024). Functional genomics in inborn errors of immunity. Immunol Rev.

[6] Hassan W., Duarte A.E. (2024). Bibliometric analysis: a few suggestions. Curr Probl Cardiol.

[7] Xiao N., Huang X., Zang W., Kiselev S., Bolkov M.A., Tuzankina I.A. (2024). Health-related quality of life in patients with inborn errors of immunity: a bibliometric analysis. Front Immunol.

[8] Aria M., Cuccurullo C. (2017). Bibliometrix: an R-tool for comprehensive science mapping analysis. J Informetr.

[9] Van Eck N.J., Waltman L., Ding Y., Rousseau R., Wolfram D. (2014). Measuring scholarly impact: methods and practice.

[10] Lunke S., Bouffler S.E., Patel C.V., Sandaradura S.A., Wilson M., Pinner J. (2023). Integrated multi-omics for rapid rare disease diagnosis on a national scale. Nat Med.

[11] Similuk M.N., Yan J., Ghosh R., Oler A.J., Franco L.M., Setzer M.R. (2022). Clinical exome sequencing of 1000 families with complex immune phenotypes: toward comprehensive genomic evaluations. J Allergy Clin Immunol.

[12] Wojcik M.H., Lemire G., Berger E., Zaki M.S., Wissmann M., Win W. (2024). Genome sequencing for diagnosing rare diseases. N Engl J Med.

[13] Kernohan K.D., Boycott K.M. (2024). The expanding diagnostic toolbox for rare genetic diseases. Nat Rev Genet.

[14] IJspeert H., Edwards E.S.J., O’Hehir R.E., Dalm V.A.S.H., van Zelm M.C. (2025). Update on inborn errors of immunity. J Allergy Clin Immunol.

[15] Abolhassani H., Avcin T., Bahceciler N., Balashov D., Bata Z., Bataneant M. (2022). Care of patients with inborn errors of immunity in thirty J Project countries between 2004 and 2021. Front Immunol.

[16] Ong M.S., Rider N.L., Stein S., Maglione P.J., Galbraith A., DiGiacomo D.V. (2024). Racial and ethnic disparities in early mortality among patients with inborn errors of immunity. J Allergy Clin Immunol.

[17] Udemgba C., Sarkaria S.K., Gleeson P., Bryant-Stephens T., Ogbogu P.U., Khoury P. (2023). New considerations of health disparities within allergy and immunology. J Allergy Clin Immunol.

[18] Kindle G., Alligon M., Albert M.H., Buckland M., Edgar J.D., Gathmann B. (2025). Inborn errors of immunity: manifestation, treatment, and outcome—an ESID registry 1994-2024 report on 30,628 patients. J Hum Immun.

[19] Maródi L., J Project Study Group (2011). The creation and progress of the J Project in Eastern and Central Europe. Ann N Y Acad Sci.

[20] Dinur-Schejter Y., Stepensky P. (2022). Social determinants of health and primary immunodeficiency. Ann Allergy Asthma Immunol.

[21] Wheeden K., Meyers S., Anthony K., Chehade M., Gifford R.C., King E.C. (2025). Enhancing and leveraging principal investigator and patient advocacy group collaboration in rare disease clinical research: meeting report from the Rare Diseases Clinical Research Network. Ther Adv Rare Dis.

[22] Zanello G., Chan C.H., Parker S., Julkowska D., Pearce D.A. (2024). Fostering the international interoperability of clinical research networks to tackle undiagnosed and under-researched rare diseases. Front Med.

[23] Klein T.L., Bender J., Bolton S., Collin-Histed T., Daher A., De Baere L. (2024). A rare partnership: patient community and industry collaboration to shape the impact of real-world evidence on the rare disease ecosystem. Orphanet J Rare Dis.

[24] Rogic S., Poirier-Morency G., Hieter P., Pavlidis P. (2025). Global partnerships in rare disease research. Dis Model Mech.

[25] Degen C. (2024).

[26] Al-Saud B., Al-Mousa H., Al Gazlan S., Al-Ghonaium A., Arnaout R., Al-Seraihy A. (2015). Primary immunodeficiency diseases in Saudi Arabia: a tertiary care hospital experience over a period of three years (2010-2013). J Clin Immunol.

[27] Al-Mousa H., Al-Dakheel G., Jabr A., Elbadaoui F., Abouelhoda M., Baig M. (2018). High incidence of severe combined immunodeficiency disease in Saudi Arabia detected through combined T cell receptor excision circle and next generation sequencing of newborn dried blood spots. Front Immunol.

[28] Sharma P., Jain A., Scaria V. (2021). Genetic landscape of rare autoinflammatory disease variants in Qatar and Middle Eastern populations through the integration of whole-genome and exome datasets. Front Genet.

[29] Aamer W., Al-Maraghi A., Syed N., Gandhi G.D., Aliyev E., Al-Kurbi A.A. (2024). Burden of Mendelian disorders in a large Middle Eastern biobank. Genome Med.

[30] Marzouka N.A.D., Alnaqbi H., Al-Aamri A., Tay G., Alsafar H. (2024). Investigating the genetic makeup of the major histocompatibility complex (MHC) in the United Arab Emirates population through next-generation sequencing. Sci Rep.

[31] Aghamohammadi A., Rezaei N., Yazdani R., Delavari S., Kutukculer N., Topyildiz E. (2021). Consensus Middle East and North Africa registry on inborn errors of immunity. J Clin Immunol.

[32] Hamdi Y., Boujemaa M., Ben Aissa-Haj J., Radouani F., Khyatti M., Mighri N. (2024). A regionally based precision medicine implementation initiative in North Africa: the PerMediNA Consortium. Transl Oncol.

[33] Baris S., Abolhassani H., Massaad M.J., Al-Nesf M., Chavoshzadeh Z., Keles S. (2023). The Middle East and North Africa diagnosis and management guidelines for inborn errors of immunity. J Allergy Clin Immunol Pract.

[34] Saleh S., Dabbous O., Sullivan S.D., Ankleshwaria D., Trombini D., Toumi M. (2024). A practical approach for adoption of a hub and spoke model for cell and gene therapies in low- and middle-income countries: framework and case studies. Gene Ther.

[35] Fasseeh A.N., Korra N., Aljedai A., Seyam A., Almudaiheem H., Al-Abdulkarim H.A. (2025). Rare disease challenges and potential actions in the Middle East. Int J Equity Health.

[36] Rouce R.H., Grilley B.J. (2025). How to democratize cell and gene therapy: a global approach. Mol Ther.

[37] Abraham R.S., Butte M.J. (2021). The new “wholly trinity” in the diagnosis and management of inborn errors of immunity. J Allergy Clin Immunol Pract.

[38] Caballero-Oteyza A., Crisponi L., Peng X.P., Yauy K., Volpi S., Giardino S. (2024). GenIA, the Genetic Immunology Advisor database for inborn errors of immunity. J Allergy Clin Immunol.

[39] Subramanian S., Hutchins J.A., Lundsteen N. (2022). Bridging the gap: increasing collaboration between research mentors and career development educators for PhD and postdoctoral training success. Mol Biol Cell.

[40] Lambert W.M., Nana N., Afonja S., Saeed A., Amado A.C., Golightly L.M. (2025). Addressing structural mentoring barriers in postdoctoral training: a qualitative study. Stud Grad Postdr Educ.

[41] Scaffidi A.K., Berman J.E. (2011). A positive postdoctoral experience is related to quality supervision and career mentoring, collaborations, networking and a nurturing research environment. Higher Education.

[42] Murray C., Santangeli E., Mockler D., Townsend K., Seidel M.G., Burns S.O., ESID Clinical Working Party (2025). A scoping review of clinical management guidelines in inborn errors of immunity. J Allergy Clin Immunol.

[43] King J.R., Notarangelo L.D., Hammarström L. (2021). An appraisal of the Wilson and Jungner criteria in the context of genomic-based newborn screening for inborn errors of immunity. J Allergy Clin Immunol.

[44] Neary C.M., Ong M.S., Farmer J.R. (2025). Sociodemographic drivers of care disparity among patients with inborn errors of immunity. Curr Opin Pediatr.

[45] Martinson A.K., Chin A.T., Butte M.J., Rider N.L. (2024). Artificial intelligence and machine learning for inborn errors of immunity: current state and future promise. J Allergy Clin Immunol Pract.

[46] Rivière J.G., Soler Palacín P., Butte M.J. (2024). Proceedings from the inaugural Artificial Intelligence in Primary Immune Deficiencies (AIPID) conference. J Allergy Clin Immunol.

