# Supplementary material for: Emerging trends and research hot spots in inborn error of immunity: A bibliometric perspective
Source: J Allergy Clin Immunol Glob. 2026 Apr 15;5(4):100704. doi: 10.1016/j.jacig.2026.100704 (PMC13138166; doi:10.1016/j.jacig.2026.100704)
Supplement: Supplementary Figs E1-E6 [file mmc1.pdf]

**SUPPLEMENTARY/ ONLINE REPOSITORY**

**Figure E1-A: International Co-Authorship Network of Countries in IEI research**

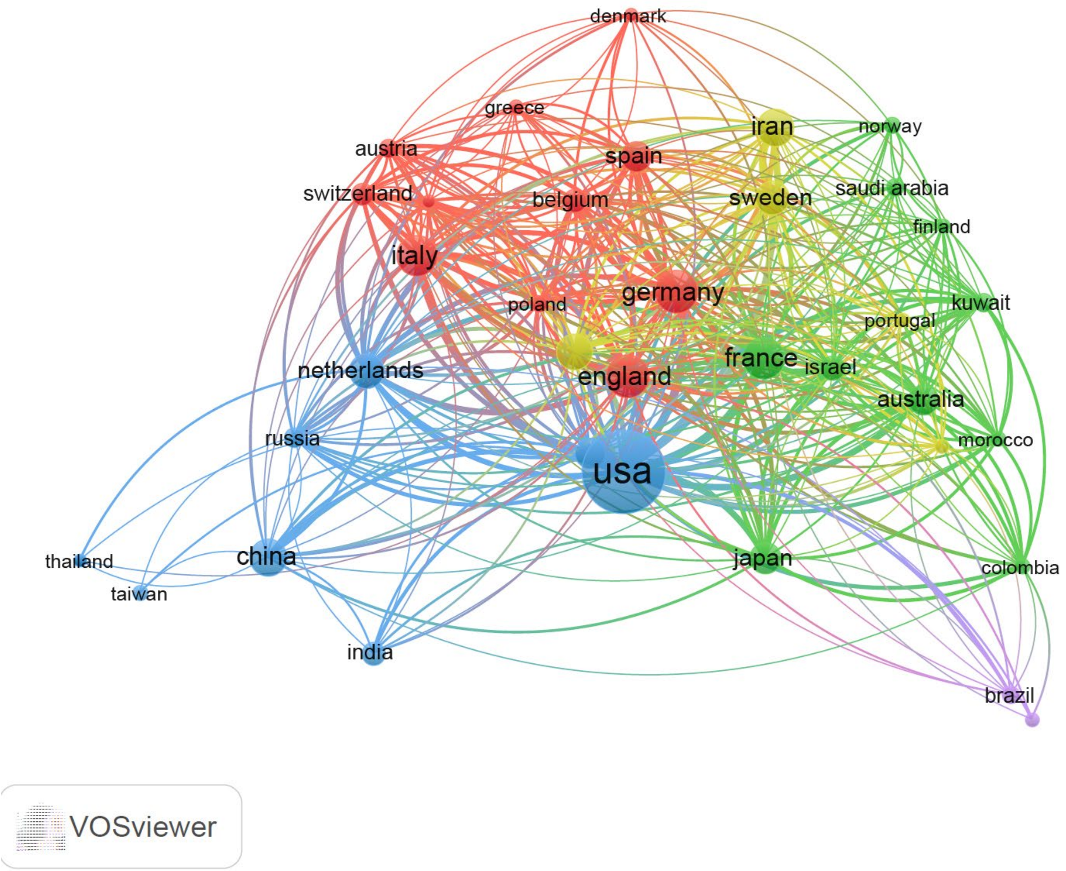


Each node represents a country, with node size proportional to the number of publications and links indicating the strength of collaborative ties. The United States, France, Germany, and the United Kingdom form central nodes, demonstrating high levels of international collaboration. Peripheral but connected countries such as Iran, China, and Brazil also show growing engagement in global research partnerships. The network highlights the collaborative and multinational nature of IEI research, where knowledge production is increasingly driven by cross-border scientific exchange.

**Figure E1-B: Temporal Overlay of International Co-Authorship in IEI Research by Country**

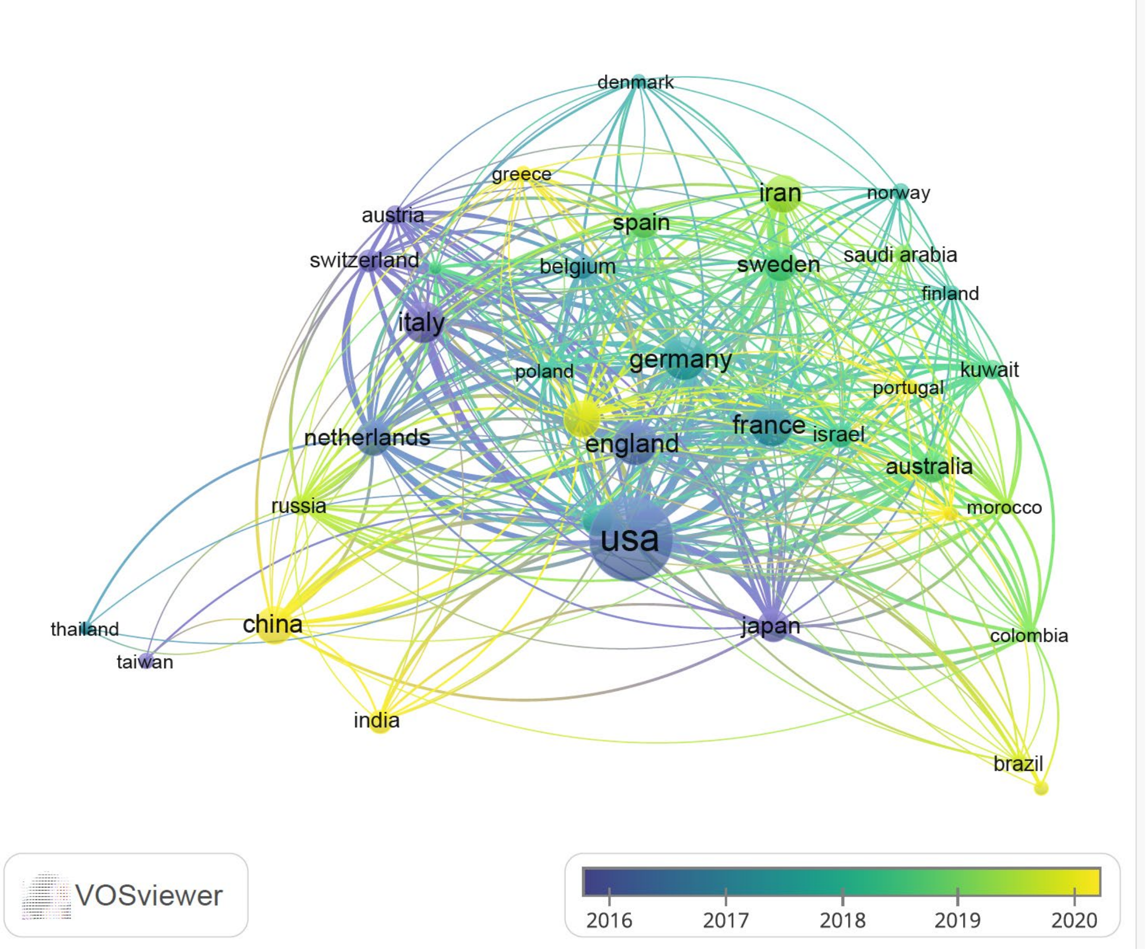


Node color indicates the average year of collaboration, with lighter colors representing more recent involvement. Countries such as Iran, China, and Brazil show increased collaborative activity in recent years, while the United States, France, and the United Kingdom maintain long-standing engagement. The map reveals both established and emerging contributors to global IEI research, reflecting the dynamic evolution of international partnerships. This temporal perspective highlights the expanding global participation and integration of newer research communities into the field.

**Figure E2-A: Institutional Co-Authorship Network in IEI Research**

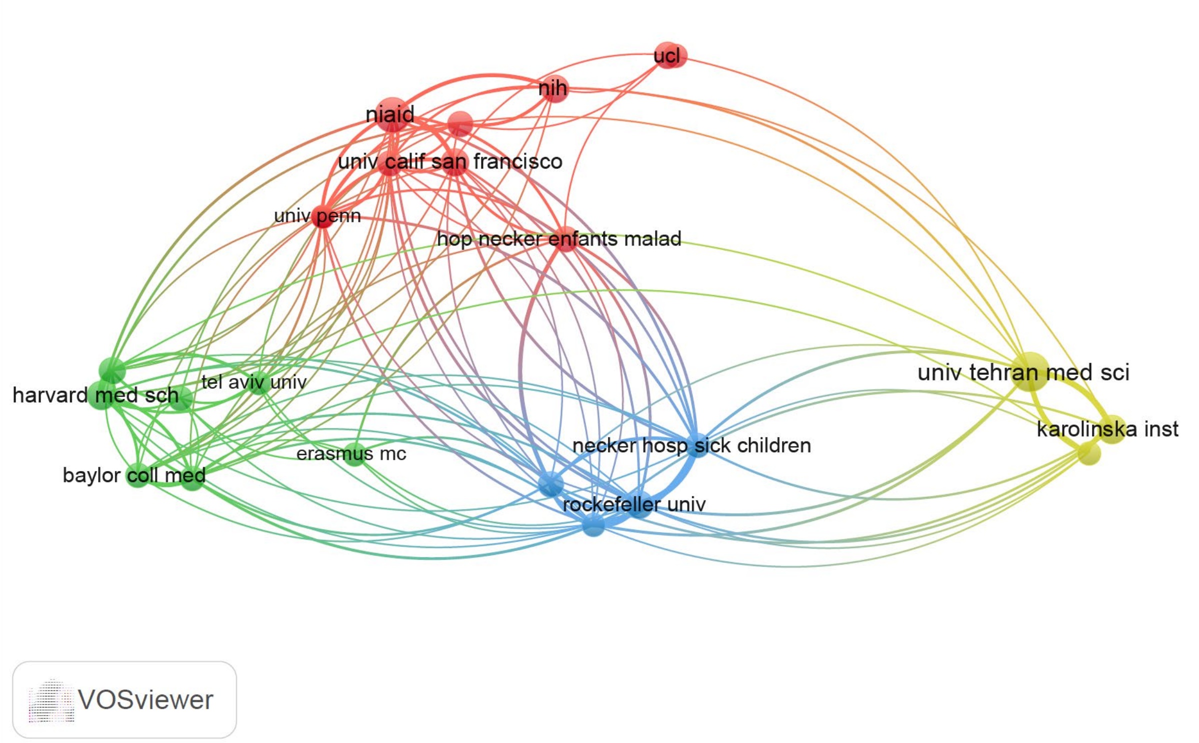


Each node represents an institution, with size indicating publication volume and links representing the frequency and strength of collaborations. Prominent institutions such as the National Institutes of Health (NIH), Harvard Medical School, Université Paris Cité (Hôpital Necker-Enfants Malades), and the University of Tehran Medical Sciences are centrally positioned, reflecting their leadership and strong collaborative activity. The map reveals clusters of institutions with dense intra-regional and inter-institutional ties, particularly across North America, Europe, and the Middle East. This network underscores the structural backbone of global IEI research, built on shared expertise and institutional partnerships.

**Figure E2-B: Temporal Overlay of Institutional Co-Authorship in IEI Research**

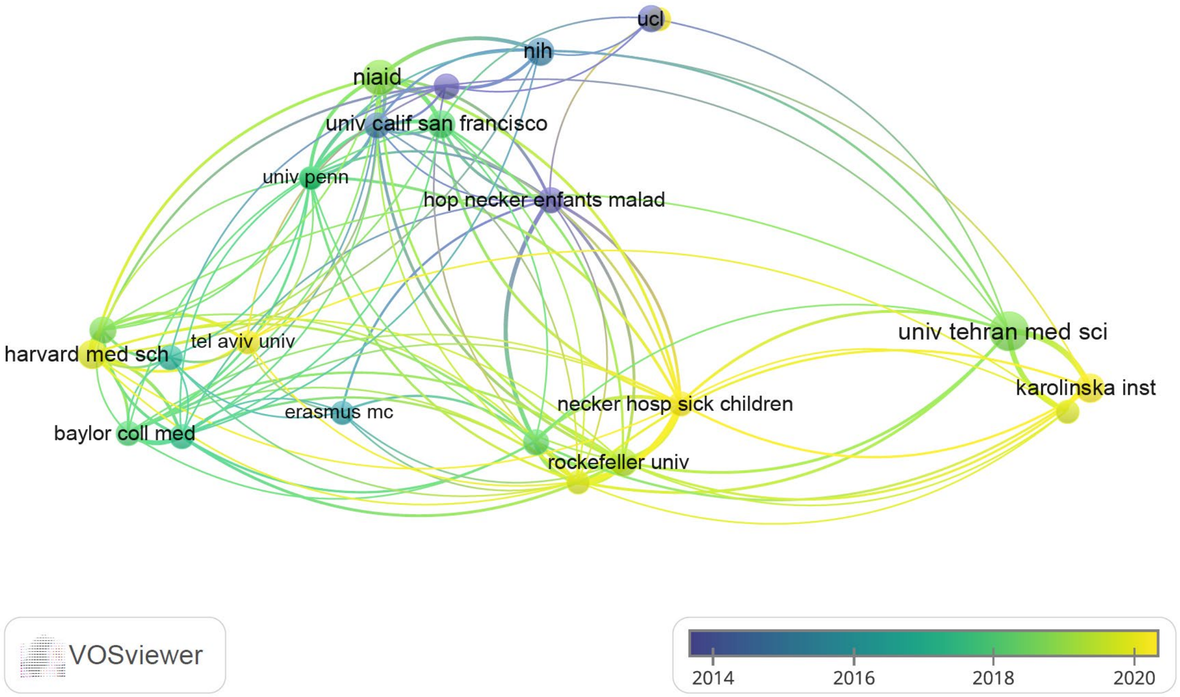


The node colors representing the average year of publication. Institutions such as the University of Tehran Medical Sciences and Erasmus MC appear in lighter shades, indicating more recent collaborative activity, while others like NIH and Harvard Medical School show longer-standing involvement. This visualization reveals both historically established contributors and emerging institutions becoming increasingly engaged in global IEI research. The evolving color gradient highlights shifts in institutional leadership and the integration of new academic centers into the collaborative landscape. It offers insight into how the field is expanding across diverse research systems over time.

**Figure E3-A: Co-authorship Network of IEI Authors**

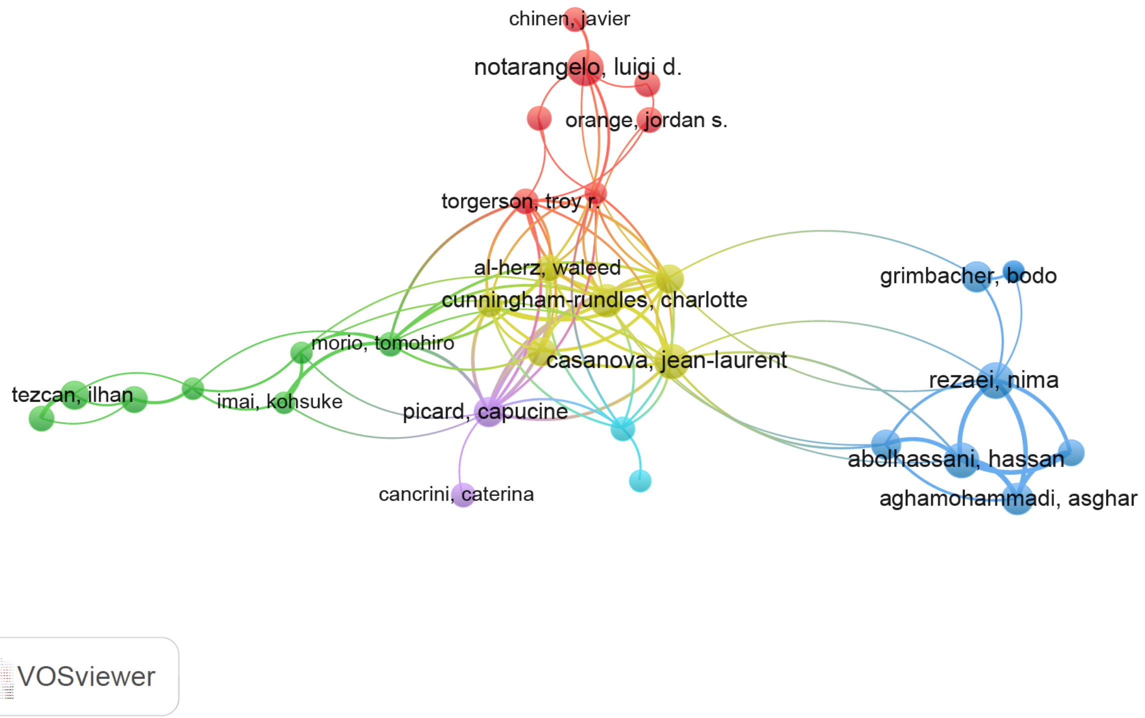


Nodes represent authors (e.g., Rezaei, Notarangelo, Picard), with size denoting publication volume and link thickness indicating co-authorship strength. The map reveals clusters reflecting strong institutional and international collaboration, particularly among leaders in clinical and genetic immunology.

**Figure E3-B: Overlay Visualization of Co-authorship Among IEI Authors**

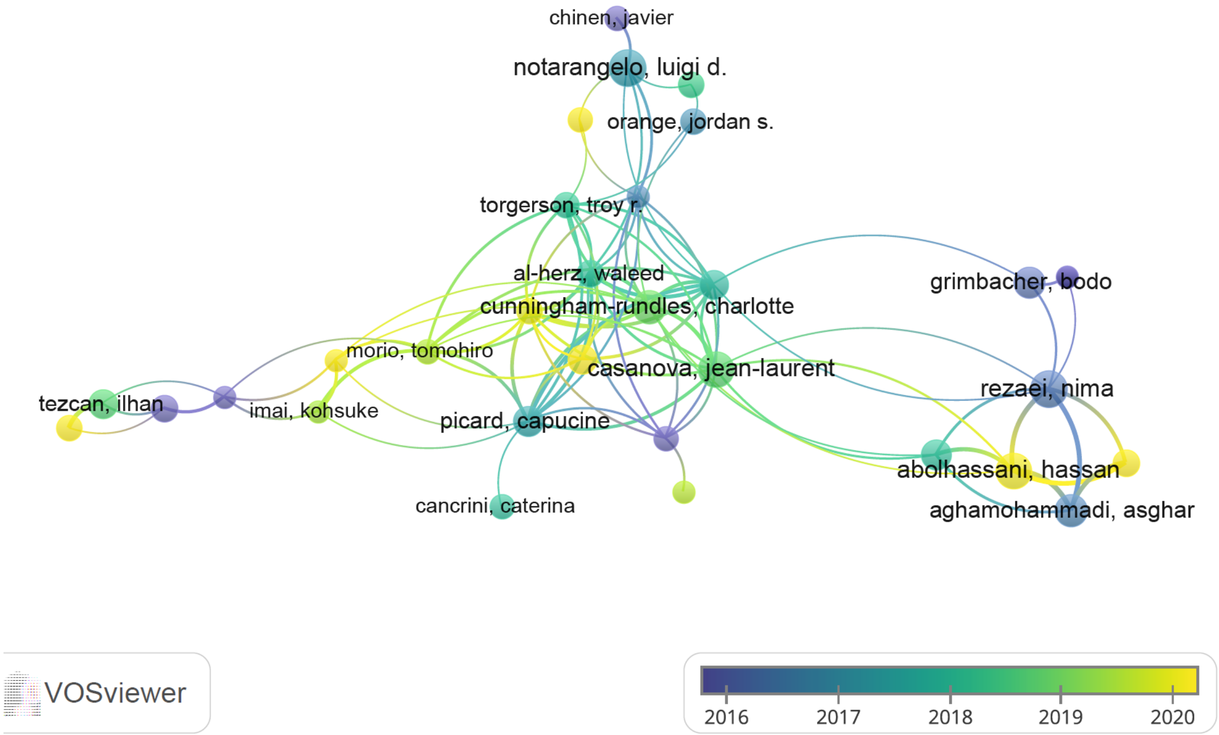


This overlay adds a temporal dimension to co-authorship links, with color gradients indicating the average publication year of each author. It shows sustained contributions by senior investigators such as Casanova and emerging collaboration patterns in recent years, highlighting evolving research dynamics in the IEI field

**Figure E3-C: Cited Author Network in IEI Research**

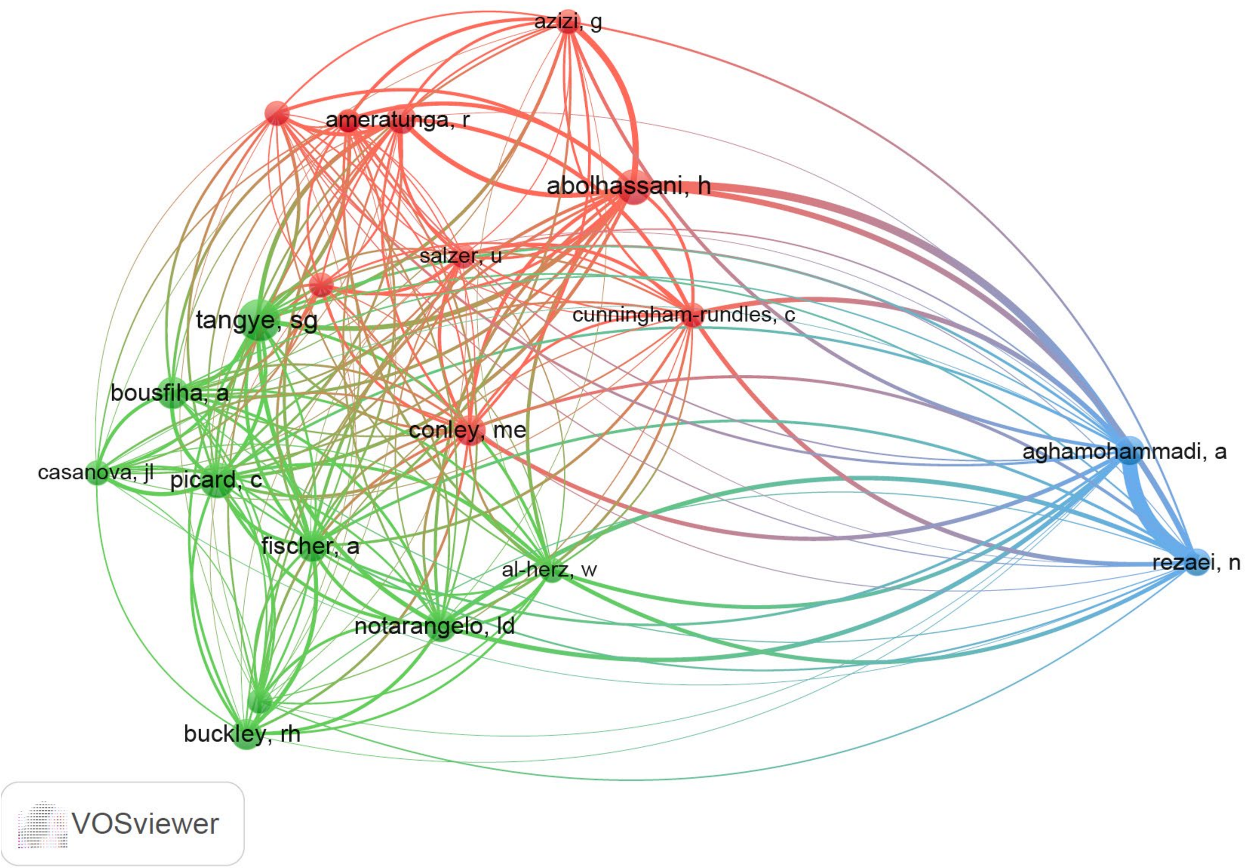


This figure illustrates the network of frequently co-cited authors, indicating those whose works are often cited together. Central nodes like Charlotte Cunningham-Rundles, Capucine Picard, and Jean-Laurent Casanova reflect foundational conceptual influence and methodological frameworks shared across IEI studies

**Figure E4-A: Citation Network of Cited Journals in IEI Research**

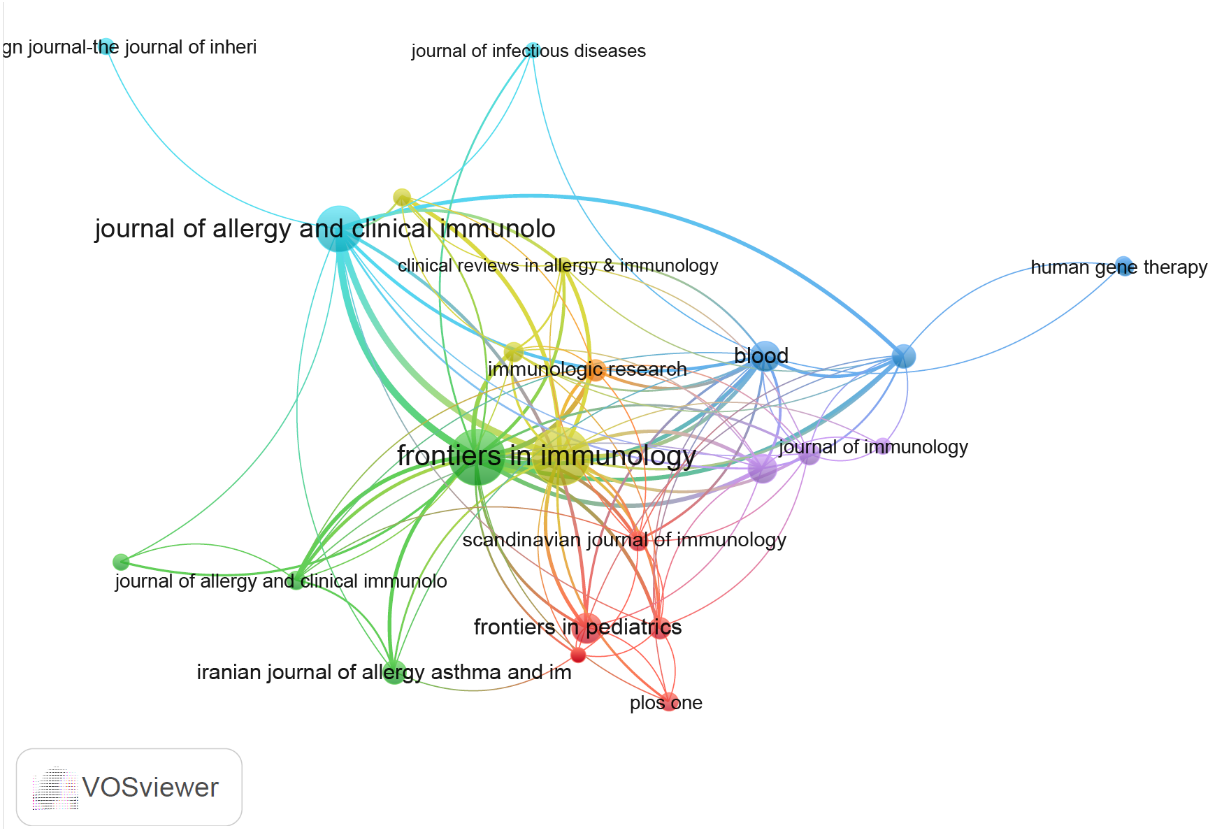


Journals that are frequently cited together are clustered by thematic proximity, revealing patterns in how IEI researchers source their knowledge. *Frontiers in Immunology*, *Blood*, and *Journal of Clinical Immunology* again emerge as major hubs, surrounded by both specialized and multidisciplinary journals. The clustering demonstrates how knowledge flows through the field, integrating immunology, genetics, and translational medicine. This structure reflects the complexity and interconnectivity of modern IEI research.

**Figure E4-B: Time-Based Mapping of Cited Journals in IEI Research**

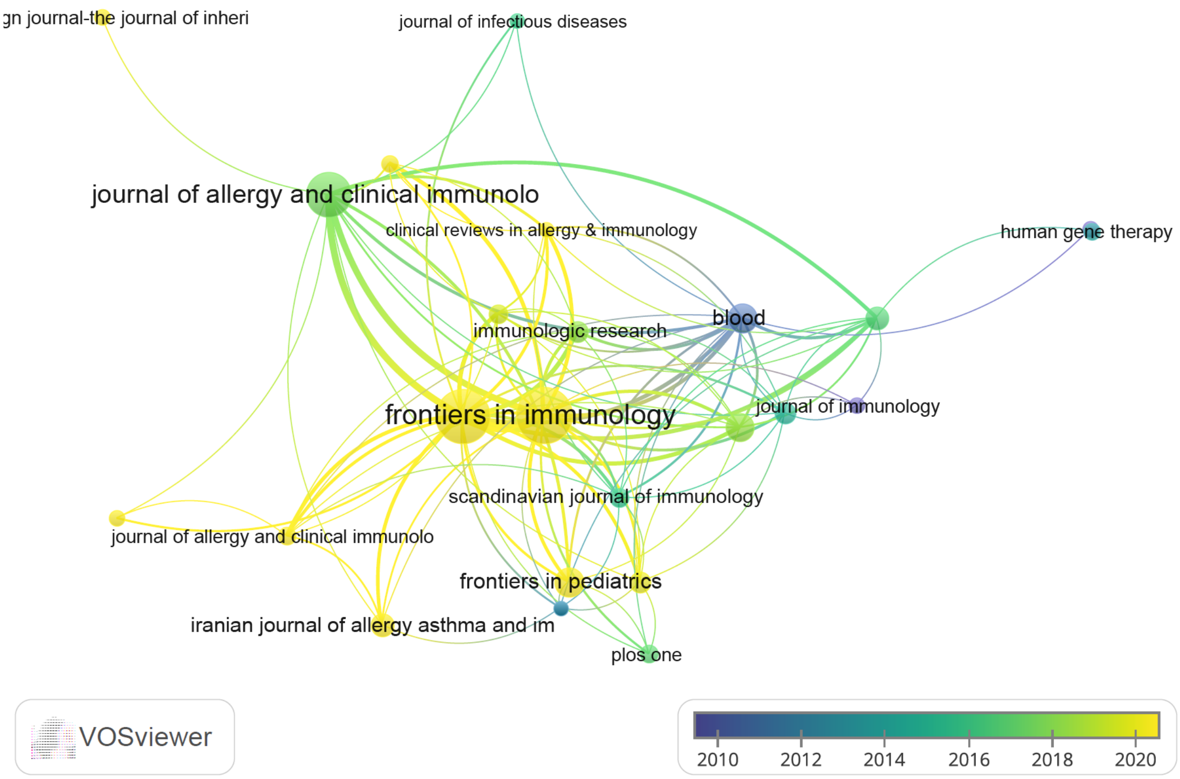


Node color indicates the average year of citation, with more recent citations shown in lighter colors. Journals like *Frontiers in Immunology* and *Journal of Allergy and Clinical Immunology* show more recent citation activity, while others reflect earlier foundational contributions. This figure provides insight into the evolution of the field, identifying which sources remain current versus those foundational in earlier decades. It supports understanding of how the citation landscape has changed over time in response to emerging discoveries.

**Figure E4-C: Co-Citation Network of Cited Journals in IEI Research**

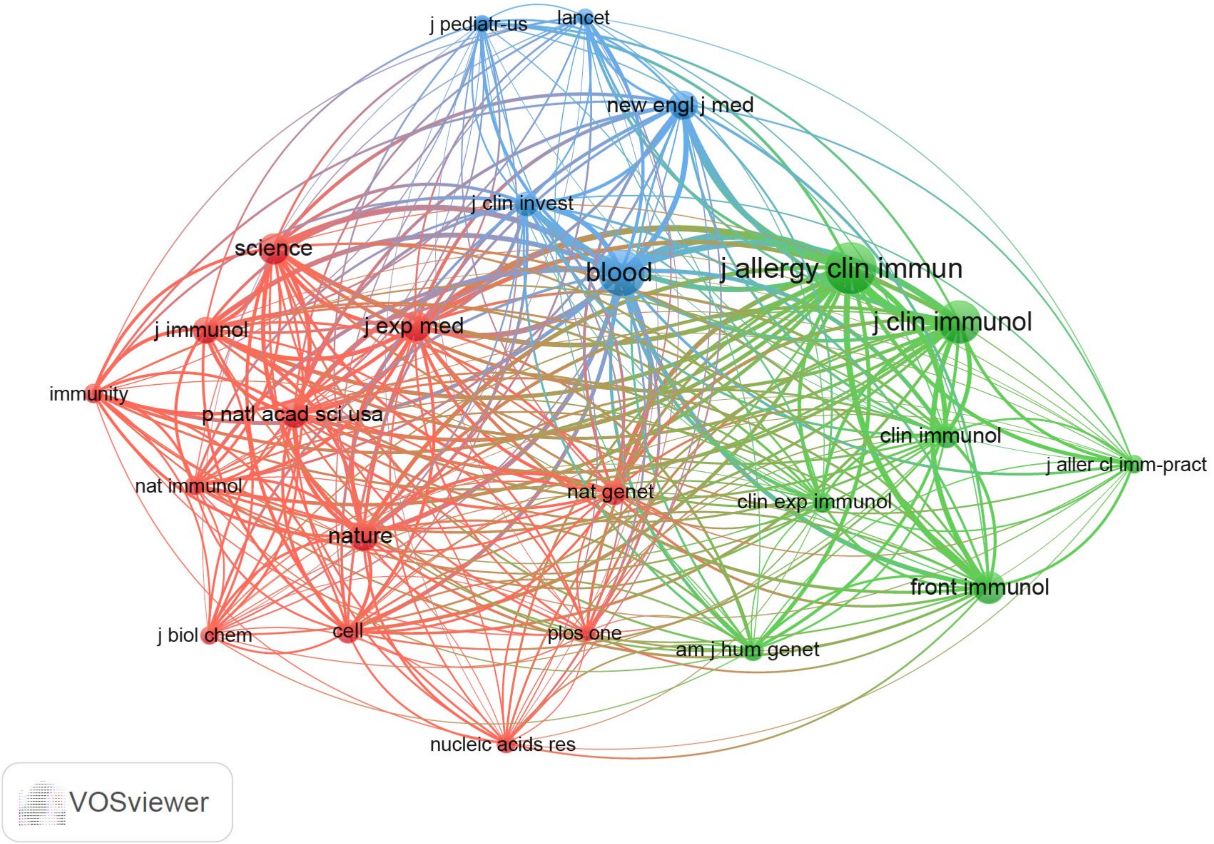


Each node represents a journal, with size indicating citation frequency and links representing co-citation strength. Core journals such as *Frontiers in Immunology*, *Journal of Clinical Immunology*, *Blood*, and *Journal of Allergy and Clinical Immunology* appear as central, densely connected nodes, indicating their foundational role in the field. High-impact general science journals like *Nature*, *Science*, and *NEJM* also appear, reflecting the interdisciplinary relevance of IEI research. The network structure highlights how clinical and molecular journals are jointly cited, suggesting a strong translational dimension.

**Figure E5: Co-Citation Network of Foundational References in IEI Literature**

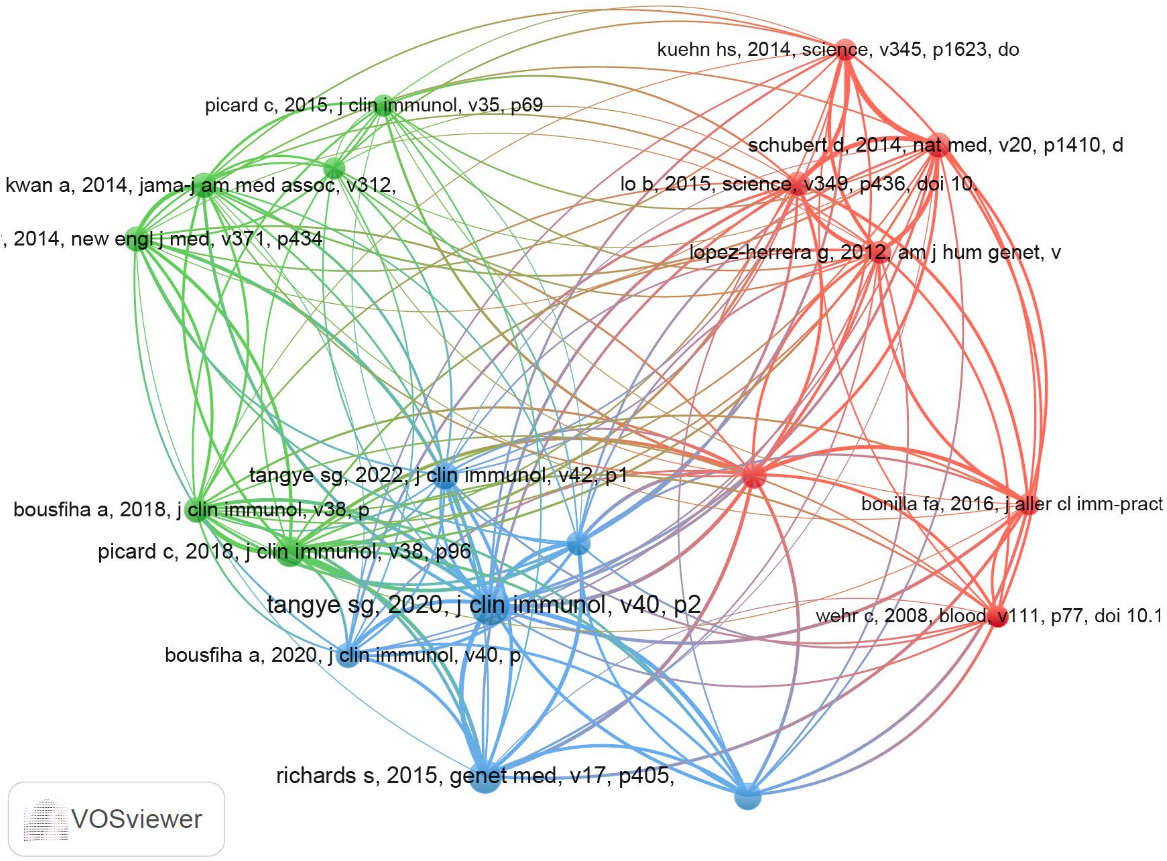


References such as the IUIS classification reports (Tangye, Picard, Bousfiha), SCID outcomes (Pai), and variant interpretation guidelines (Richards) are centrally positioned, indicating wide citation across multiple studies. The presence of mechanistic studies (e.g., CTLA4 variants) alongside clinical diagnostic frameworks emphasizes the integration of molecular and clinical paradigms. This figure helps define the intellectual backbone of the field, guiding diagnostic, therapeutic, and research priorities in IEI.

**Figure E6-A: Network Visualization of Keywords in IEI Research**

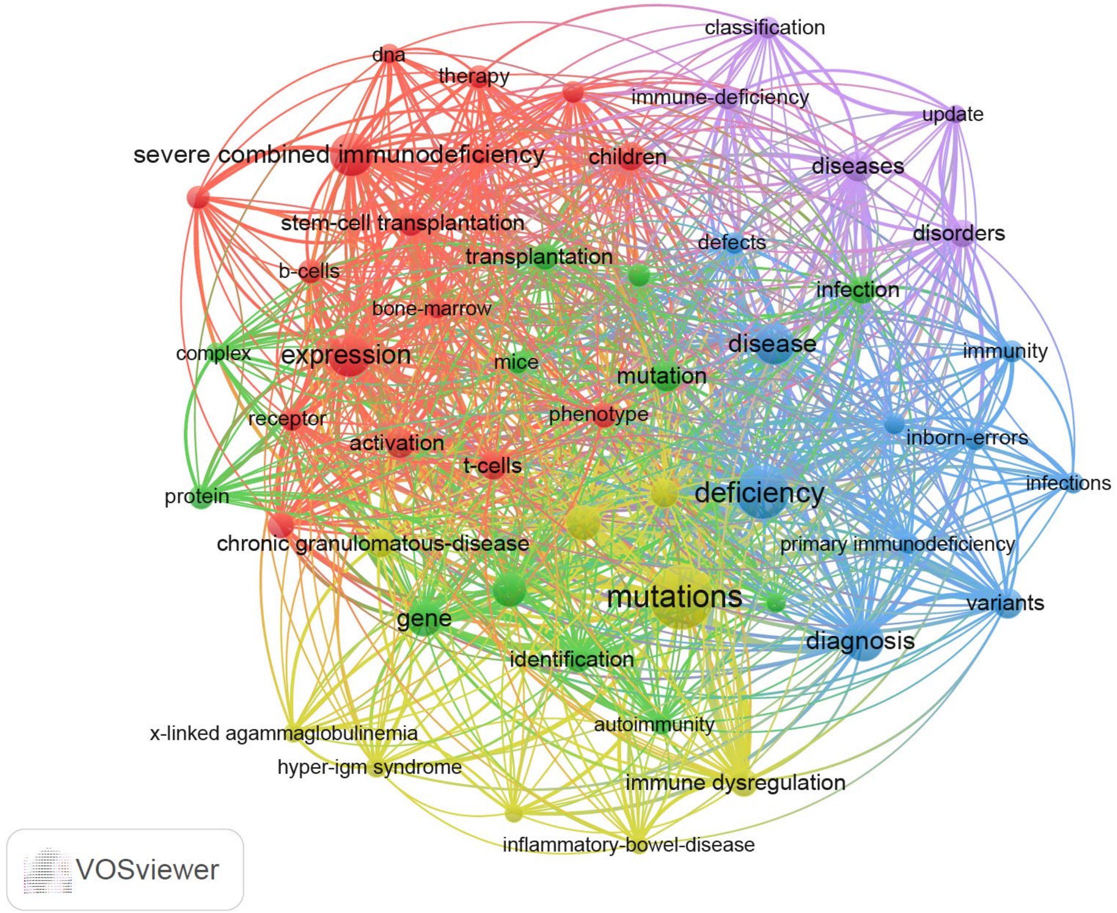


This co-occurrence network groups author keywords by thematic similarity. Terms such as "primary immunodeficiency," "gene," "mutation," and "autoimmunity" cluster together, demonstrating the interconnected focus on clinical presentation and molecular underpinnings. The density of connections reflects the interdisciplinary nature of IEI research bridging diagnostics, therapy, and genetic mechanisms.

**Figure 6-B: Overlay Visualization of Keywords by Publication Year**

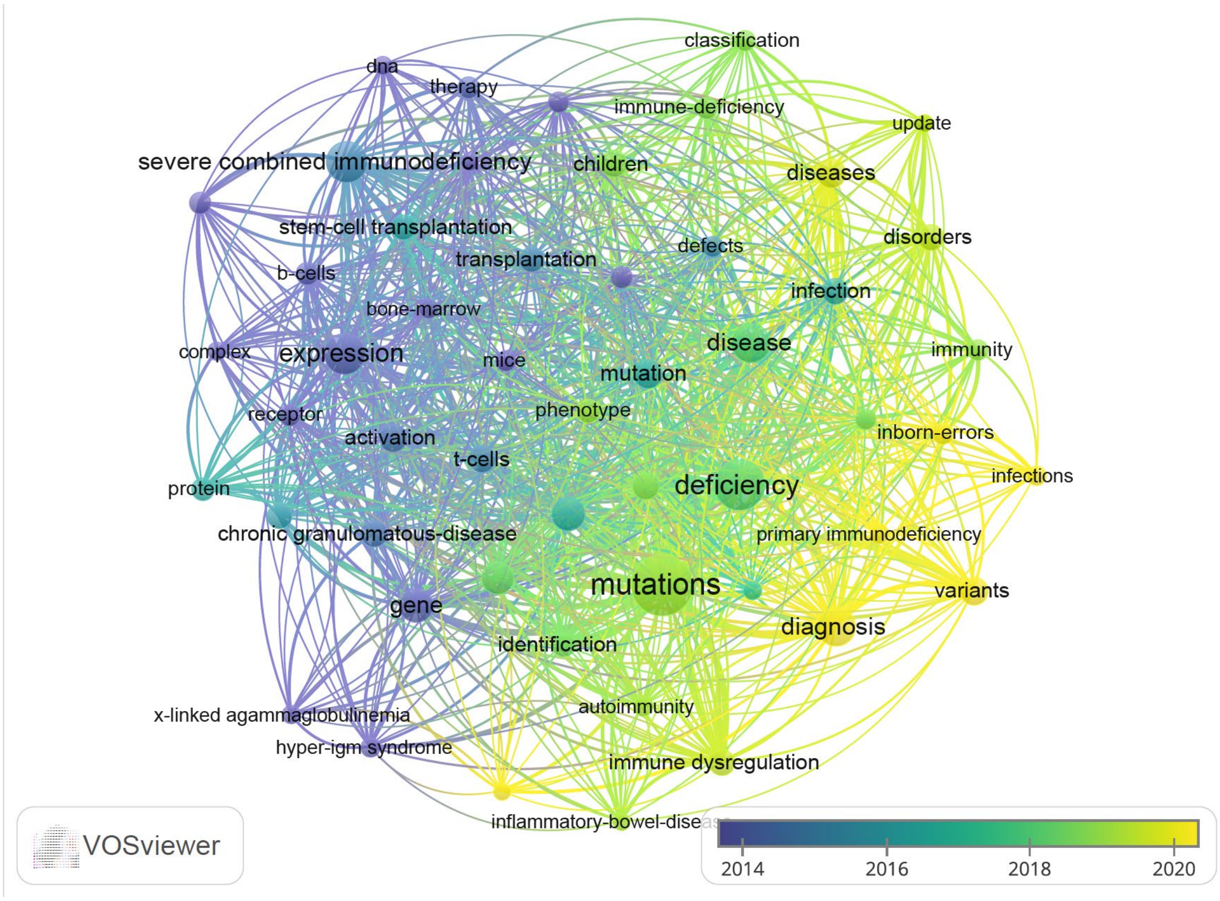


This overlay map shows the temporal emergence of research themes. Newer topics like "whole exome sequencing," "genetic testing," and "inborn error of immunity" appear in lighter colors, indicating recent emphasis. Earlier terms such as "primary immunodeficiency" and "stem-cell transplantation" remain foundational. The visual progression captures the evolution of IEI research toward genomics-driven approaches.
