# Supplementary material for: Emerging trends and research hot spots in inborn error of immunity: A bibliometric perspective
Source: J Allergy Clin Immunol Glob. 2026 Apr 15;5(4):100704. doi: 10.1016/j.jacig.2026.100704 (PMC13138166; doi:10.1016/j.jacig.2026.100704)
Supplement: Supplementary Tables E1-E4 [file mmc2.pdf]

SUPPLEMENTARY/ ONLINE REPOSITORY

Table E1: Retrieval strategy

	Search Criteria	Records
**Records were identified through the WoSCC database**	#1: (TI=("genetic defect*" OR "genetic mutation*" OR "genetic variant*" OR "monogenic disorder*" OR "hereditary mutation*" OR "inherited mutation*" OR "pathogenic variant*" OR "genetic disease*" OR "monogenic disease*" OR "genomic alteration*" OR "gene defect*") OR AB=("genetic defect*" OR "genetic mutation*" OR "genetic variant*" OR "monogenic disorder*" OR "hereditary mutation*" OR "inherited mutation*" OR "pathogenic variant*" OR "genetic disease*" OR "monogenic disease*" OR "genomic alteration*" OR "gene defect*"))<br><br>#2: (TI=("severe combined immune deficienc*" OR "SCID" OR "severe combined immunodeficienc*" OR "common variable immunodeficienc*" OR "IEI" OR "inborn errors of immunit*" OR "primary immunodeficienc*" OR "PID" OR "immunodeficiency disorder*" OR "inherited immunodeficienc*" OR "congenital immunodeficienc*" OR "rare immune disorder*" OR "rare immune disease*") OR AB=("severe combined immune deficienc*" OR "SCID" OR "severe combined immunodeficienc*" OR "common variable immunodeficienc*" OR "IEI" OR "inborn errors of immunit*" OR "primary immunodeficienc*" OR "PID" OR "immunodeficiency disorder*" OR "inherited immunodeficienc*" OR "congenital immunodeficienc*" OR "rare immune disorder*" OR "rare immune disease*"))	
**Time span (index date): 1995.01.15 to 2025.01.15** (The search was conducted on Jan 16, 2025)<br><br>**Languages: English**	# 3 = #1 AND #2	**1,236**<br><br>**1,201**
**Excluded Literature**	Review Article (n=354), Book Chapters (n=22), Proceeding Paper (n=21), Meeting Abstract (n=19), Editorial Material (n=11), Letter (n=3), News item (n=1), Retracted Publication (n=1), Excluded by Expert (n = 19)	**451**
**Literature added from PubMED database**	Records were identified through the PubMED database using same Search Criteria:<br>- Articles (n = 1384)<br>- English Language (n = 1317)<br>- Screened by Expert (n = 654)<br>- Not available in WoSCC (n = 24)	**630**
**Remaining Publications**	**210** Unique Articles from WoSCC,<br>**90** Unique Articles from PubMED,<br>**540** Same Articles from both WoSCC and PubMED.	**840**

Table E2: Top 10 countries contributing to IEI research

Rank	Country	Counts	Citations	Average Citation/ Publications	Organization (Country)	Counts
1	United States	1307	10105	48.6	Universite Paris Cite (France)	275
2	Iran	507	609	11.7	Institut National De La Sante Et De La Recherche Medicale (INSERM) (France)	169
3	France	470	1702	54.9	Assistance Publique Hopitaux Paris (APHP) (France)	162
4	Germany	415	3000	68.2	National Institutes of Health (NIH) (USA)	155
5	Italy	289	632	14.7	Tehran University of Medical Sciences (Iran)	138
6	England	278	3182	77.6	Harvard University (USA)	135
7	Spain	264	513	19	Hopital Universitaire Necker-Enfants Malades - APHP (France)	101
8	China	233	497	8.6	University of Freiburg (Germany)	100
9	Japan	231	436	15.6	University of California System (USA)	99
10	Netherlands	190	1600	53.3	University of London (UK)	92

This table presents the top 10 countries contributing to IEI research, ranked by publication volume, along with their total citation counts and average citations per publication. It also includes the top 10 most productive institutions, based on their number of IEI-related publications.

Table E3: Top Authors and Co-Cited Contributors in IEI Research

Rank	Author	Counts	Citations	H-index	Co-cited Authors	Citations
1	Rezaei, Nima	27	1523	14	Picard, Capucine	287
2	Notarangelo, Luigi D.	27	719	13	Cunningham-rundles, Charlotte	285
3	Abolhassani, Hassan	27	603	13	Al-Herz, Waleed	250
4	Casanova, Jean-Laurent	22	2988	13	Klein, Christoph	249
5	Picard, Capucine	21	4261	17	Bousfiha, Aziz	227
6	Aghamohammadi, Asghar	21	508	12	Sullivan, Kathleen E.	226
7	Grimbacher, Bodo	19	1923	12	Holland, Steven M.	225
8	Hammarstrom, Lennart	16	518	10	Franco, Jose Luis	217
9	Klein, Christoph	15	3092	12	Casanova, Jean-Laurent	216
10	Cunningham-rundles, Charlotte	15	2825	10	Oksenhendler, Eric	203

The top 10 most prolific authors in IEI research based on publication count, along with total citations and H-index (based on number of papers extracted). It also lists the most frequently co-cited authors, reflecting those whose work has significantly influenced the field, even if not directly among the most published.

Table E4: Frequently Co-Cited References in IEI Research

Rank	Co-cited Reference	Citations	Title	PubMED ID
1	tangye sg, 2020, j clin immunol, v40, p24, doi 10.1007/s10875-019-00737-x	100	Human Inborn Errors of Immunity: 2019 Update on the Classification from the International Union of Immunological Societies Expert Committee	31953710
2	richards s, 2015, genet med, v17, p405, doi 10.1038/gim.2015.30	71	Standards and guidelines for the interpretation of sequence variants: a joint consensus recommendation of the American College of Medical Genetics and Genomics and the Association for Molecular Pathology	25741868
3	picard c, 2018, j clin immunol, v38, p96, doi 10.1007/s10875-017-0464-9	57	International Union of Immunological Societies: 2017 Primary Immunodeficiency Diseases Committee Report on Inborn Errors of Immunity	29226302
4	tangye sg, 2022, j clin immunol, v42, p1473, doi 10.1007/s10875-022-01289-3	48	Human Inborn Errors of Immunity: 2022 Update on the Classification from the International Union of Immunological Societies Expert Committee	35748970
5	seidel mg, 2019, j aller cl imm-pract, v7, p1763, doi 10.1016/j.jaip.2019.02.004	45	The European Society for Immunodeficiencies (ESID) Registry Working Definitions for the Clinical Diagnosis of Inborn Errors of Immunity	30776527
6	kwan a, 2014, jama-j am med assoc, v312, p729, doi 10.1001/jama.2014.9132	40	Newborn screening for severe combined immunodeficiency in 11 screening programs in the United States	25138334
7	bousfiha a, 2018, j clin immunol, v38, p129, doi 10.1007/s10875-017-0465-8	39	The 2017 IUIS Phenotypic Classification for Primary Immunodeficiencies	29226301
8	bousfiha a, 2020, j clin immunol, v40, p66, doi 10.1007/s10875-020-00758-x	39	Human Inborn Errors of Immunity: 2019 Update of the IUIS Phenotypical Classification	32048120
9	pai sy, 2014, new engl j med, v371, p434, doi 10.1056/nejmoa1401177	39	Transplantation outcomes for severe combined immunodeficiency, 2000-2009	25075835
10	schubert d, 2014, nat med, v20, p1410, doi 10.1038/nm.3746	39	Autosomal dominant immune dysregulation syndrome in humans with CTLA4 mutations	25329329

The top 10 most frequently co-cited references in IEI research, including citation counts, titles, and PubMed IDs. These references represent key classification frameworks, diagnostic guidelines, and landmark studies that form the conceptual backbone of the field.
